# Mitigating the impact of conference and travel cancellations on researchers’ futures

**DOI:** 10.7554/eLife.57032

**Published:** 2020-03-27

**Authors:** Tracey Weissgerber, Yaw Bediako, Charlotte M de Winde, Hedyeh Ebrahimi, Florencia Fernández-Chiappe, Vinodh Ilangovan, Devang Mehta, Carolina Paz Quezada, Julia L Riley, Shyam M Saladi, Sarvenaz Sarabipour, Andy Tay

**Affiliations:** 1 QUEST – Quality | Ethics | Open Science | Translation, Charité – Universitätsmedizin Berlin, Berlin Institutes of HealthBerlinGermany; 2 Division of Nephrology and Hypertension, Mayo ClinicRochesterUnited States; 3 West African Centre for Cell Biology of Infectious Pathogens, University of GhanaAccraGhana; 4 MRC Laboratory for Molecular Cell Biology, University College LondonLondonUnited Kingdom; 5 Non-Communicable Diseases Research Center, Endocrinology and Endocrinology and Metabolism Population Sciences Institute, Tehran University of Medical SciencesTehranIslamic Republic of Iran; 6 Instituto de Investigación en Biomedicina de Buenos Aires - CONICET - Partner Institute of the Max Planck SocietyBuenos AiresArgentina; 7 Aarhus UniversityAarhusDenmark; 8 Department of Biological Sciences, University of AlbertaEdmontonCanada; 9 Centro Integrativo de Biología y Química Aplicada, Universidad Bernardo O'HigginsSantiagoChile; 10 Department of Botany and Zoology, Stellenbosch UniversityStellenboschSouth Africa; 11 Department of Biology, Dalhousie UniversityHalifaxCanada; 12 Biochemistry and Molecular Biophysics Option, California Institute of TechnologyPasadenaUnited States; 13 Institute for Computational Medicine and the Department of Biomedical Engineering, Johns Hopkins UniversityBaltimoreUnited States; 14 Department of Biomedical Engineering, National University of SingaporeSingaporeSingapore

**Keywords:** early-career researchers, COVID-19, conferences, research assessment, science funding, travel

## Abstract

The need to protect public health during the current COVID-19 pandemic has necessitated conference cancellations on an unprecedented scale. As the scientific community adapts to new working conditions, it is important to recognize that some of our actions may disproportionately affect early-career researchers and scientists from countries with limited research funding. We encourage all conference organizers, funders and institutions who are able to do so to consider how they can mitigate the unintended consequences of conference and travel cancellations and we provide seven recommendations for how this could be achieved. The proposed solutions may also offer long-term benefits for those who normally cannot attend conferences, and thus lead to a more equitable future for generations of researchers.

The current novel coronavirus pandemic (COVID-19) is a global health emergency and the situation is rapidly changing, in most places for the worse. Everyone’s priority right now must be to support efforts to slow the spread of the outbreak, to protect themselves and those around them, and to help those who have been affected and their families. We should all also, as much as is possible, remain mindful of the unintended consequences of our actions.

The scientific community has taken important steps in response to the growing pandemic, all while experiencing widespread disruptions. Clinicians are on the front lines, focused on caring for patients, while researchers with relevant expertise are helping to better understand the virus and devise ways to protect against it. Courses and meetings have been moved online and laboratories have closed. Scientists are working from home, often with children; others may be ill or caring for loved ones who are sick. Conference organizers and societies have canceled meetings on an unprecedented scale (Robins, 2020; Service, 2020), and attendance at conferences that are still going ahead is likely to be lower than expected as researchers cancel their travel plans, either voluntarily or due to imposed restrictions.

As scientists, we strongly support the difficult decisions that have been made to protect public health. We encourage conferences that have not yet decided to cancel upcoming meetings to do so well in advance, in line with public health recommendations (Centers for Disease Control and Prevention, 2020). As advocates for early-career researchers (ECRs), we are also concerned with how the repercussions of these vitally necessary actions may disproportionately affect early-career scientists and researchers from countries with limited research funding.

Researchers often see conferences and other in-person meetings as an investment in their future. Conferences are valuable opportunities to gain recognition for, and feedback on, one’s work, and to establish connections with potential colleagues and collaborators from around the world. For this reason, we encourage all conference organizers, research funders and institutions who are able to do so to consider steps that we believe would help mitigate the impacts of conference and travel cancellations. This includes both the financial impacts and the lost career opportunities. We recognize that implementation will be challenging and that meeting cancellations are only one of many disruptions currently affecting the research community. Nevertheless, we ask those who have the time and resources available to consider the following seven recommendations (Box 1) to lessen the consequences of conference and travel cancellations on the most vulnerable researchers in our communities. For those who are unable to take these actions now, we would ask that they take time to consider them after the situation has returned to some semblance of normality.

Box 1. Recommendations to help mitigate the impacts of conference and travel cancellations on researchers.

1. Conference organizers should promptly issue refunds and prioritize those experiencing financial hardship
2. Funders should reimburse researchers for canceled travel, even if the cancellation is voluntary
3. Institutions could help cover remaining expenses
4. Organizers could make conferences virtual rather than canceling or postponing
5. Conferences that are not canceled can be livestreamed to enable remote participation
6. Organizers should facilitate online networking events
7. The community should recognize canceled talks, papers and abstracts as ‘accepted for presentation’ on CVs

## Prioritize refunds

We recognize that conference cancellations may have a severe impact on organizations’ and societies’ finances, though we ask that they endeavor to issue refunds of registration fees promptly. If needed, refunds should be prioritized for those researchers who are most likely to be experiencing financial hardship in this difficult time. Researchers from countries with limited funding, graduate students and postdoctoral researchers tend to be the lowest paid and are perhaps more likely to have covered the costs of attending these meetings out of their own pocket (Malloy, 2020). These groups’ risk of financial hardship may be exacerbated by the fact that those with limited resources are often obligated to book the cheapest flights and accommodation, which may not be eligible for refunding or rebooking. For meetings that are not canceled, refunds of registration fees should be given to those who are unable to attend due to travel bans, or who choose not to attend for reasons of personal safety and public health.

We ask that any society or organization that is currently facing financial difficulties still give registrants the option to request a refund or, where applicable, choose to have their fee ‘roll forward’ for next year’s event (Hines, 2020). We would advise those in this situation to be transparent about the expected impact on their finances; some registrants may choose to donate the money to keep the organization afloat.

## Reimburse all costs associated with canceled travel

Funders should allow researchers to obtain reimbursement for canceled travel, even if the cancellation is voluntary. Agencies such as the Canadian Tri-Council have recently adopted such measures and may serve as an example (Fraser and Sauvé-McCuan, 2020). Funders who have already awarded travel grants to ECRs or scientists from countries with limited research funding could allow the recipients to defer the use of these funds for an additional year. In addition, visa fees can be prohibitively expensive, disproportionately impact researchers from countries with limited research funding, and often are not covered by funding agencies and travel grants. We therefore urge funders that do not currently cover visa fees to consider revising these policies, and to do so retroactively to assist researchers facing financial hardship due to conference and travel cancellations.

## Cover remaining expenses

Institutions can help by reimbursing ECRs for expenses that will not be covered by conference organizers or funding agencies. ECRs who paid in advance may find that registration fees will not be refunded, that their funding agency or travel grant will not allow reimbursement, or that their travel arrangements cannot be canceled. Institutions may also consider offering advances to ECRs who are awaiting refunds or reimbursement from conference organizers, travel providers and funding agencies. Obtaining refunds may be a lengthy process, as many organizations lack clear policies and staff to handle large-scale cancellations. Providing advances on refunds and reimbursements will reduce stress and financial hardships for ECRs with limited resources.

## Go virtual

While established researchers are more likely to have symposium talks that can be carried forward to the next year, early-career researchers often present research projects that may be published before the next conference. Canceling or postponing meetings may cost ECRs their one opportunity to present their research. For this reason, we ask that conference organizers consider making canceled in-person conferences virtual instead. For example, talks could be given via videoconferencing and poster sessions could be hosted online (Table 1).

**Table 1.** Ideas for virtual conferences.

Option	Example(s)
Organize virtual poster sessions with live video and chat options.	Cognitive Neuroscience Society (2020)
Make abstracts, posters, and other conference materials freely available online for anyone to read and share.	SOT 2020 (Hines, 2020)
Allow speakers to offer a pre-recorded talk for any online session. Provide instructions for recording a talk. Invite pre-recorded speakers for a live Q and A session after their virtual talk, at a time that accommodates the speaker’s time zone.	Conference on Retroviruses and Opportunistic Infections (CROI Foundation/IAS–USA, 2020)
Organize a virtual conference, followed by a virtual ‘unconference’ where scientists interact with others in small groups that are automatically matched based on similar interests.	Neuromatch 2020
Organize a free virtual conference using social media and videoconferencing.	Librarians Building Momentum for Reproducibility (2020) with draft guidance following their organizing experience available from Sayre et al. (2020) 1^st^ International Twitter Conference of Herpetology (Associação Portuguesa de Herpetologia, 2018)

This is not an exhaustive list.

We are aware that switching meetings to a virtual setting would likely place an additional burden on the conference organizers, who are already dealing with the logistical and financial impact of cancellation. One solution to this problem may be to invite ECRs and researchers from countries with limited research funding to join committees to organize the online events. This may provide those individuals with valuable career development and networking experience.

## Livestream for remote participation

Conferences that are not canceled should be livestreamed. This would allow scientists, who cannot – or no longer wish to – travel, to participate virtually. Ideally, organizers should allow new registrations from people who did not register initially because they were unable to attend in person.

## Move networking online

Conferences also provide important opportunities to network, attend workshops and interview for positions. Delaying these opportunities until next year may have long-term consequences for graduate students and postdoctoral researchers, who only have a few years to complete their projects and plan the next phase of their career. Conference organizers should therefore look into hosting virtual networking events that would allow small groups of ECRs and researchers from countries with limited research funding to interact with more senior researchers.

## Give recognition

Being selected to present at a conference is one of the ways that the scientific community recognizes the work of an individual researcher. The scientific community should agree that researchers can list conference activities on their CVs, even if the conference was canceled or the researcher was unable to attend. This is particularly important for conference activities that will not be published, though conference organizers should make abstract books freely available online, and speakers could consider sharing their slides or recordings of their talk with the community.

While we hope that these suggestions may alleviate some of the anxiety and disruption caused by COVID-19, we also encourage the scientific community to consider a global perspective. Our current reliance on in-person conferences for networking and career development creates systemic inequalities between those who have the resources to attend conferences regularly and those who do not. Many scientists in developed countries are now pausing to consider the opportunities that they will miss out on as they cancel their travel plans. As we reflect on this, it is important to remember that these experiences are routine for scientists in countries with limited research funding, as well as for those with disabilities or chronic illnesses that limit their attendance at scientific meetings.

We hope that the widespread disruptions resulting from the COVID-19 pandemic will draw attention to the challenges that our more vulnerable colleagues regularly encounter. For instance, several of the measures suggested above are necessary because ECRs are routinely required to pay for conferences and travel out of their own pocket before seeking reimbursement several months later. This is an insurmountable financial barrier for many ECRs and scientists from countries with limited research funding (Malloy, 2020). We hope that this crisis leads to widespread adoption of new policies to pay ECRs for conferences and travel costs upfront.

We also hope that the research community will learn from this experience by developing and refining alternatives to in-person conferences (Table 1). While the current emphasis is on online formats, we must also recognize that 46% of the global population has no internet connectivity (ITU, 2020). We thus need solutions that do not depend on reliable internet access as this may create further barriers for researchers who do not have high speed internet (Bohannon, 2016). This includes some scientists in countries with limited research funding, and those working in remote or rural areas. The number of scientists with difficulties accessing the internet may also increase as more people begin working from home.

In addition to helping those who cannot attend conferences due to COVID-19, new solutions could also offer long-term benefits for researchers who normally cannot attend conferences. This includes researchers who are disabled, have chronic diseases, care for family members, or researchers who experience problems caused by visa restrictions or a lack of funding. While slowing the spread of this new virus and caring for those who require medical care must remain our top priority, the current challenges we face as a scientific community may also offer new opportunities to build a more equitable global community for the scientists of tomorrow.
